# Dataset of *Sgo1* expression in cardiac, gastrointestinal, hepatic and neuronal tissue in mouse

**DOI:** 10.1016/j.dib.2017.06.046

**Published:** 2017-07-01

**Authors:** Andrew T. Song, Antonella Galli, Severine Leclerc, Stanley Nattel, Craig Mandato, Gregor Andelfinger

**Affiliations:** a Cardiovascular Genetics, Department of Pediatrics, CHU Sainte-Justine, Canada; b Wellcome Trust Sanger Institute, Hinxton, Cambridge, UK; c Université de Montréal, Montreal, QC, Canada; d McGill University, Department of Anatomy and Cell Biology, Montreal, QC, Canada; e Department of Medicine, Montreal Heart Institute and Université de Montréal, Canada; f Department of Pharmacology and Therapeutics, McGill University, Canada; g Institute of Pharmacology, West German Heart and Vascular Center, Faculty of Medicine, University Duisburg-Essen, Essen, Germany

**Keywords:** Heart development, Intestinal development, Retinal development, SGO1, CAID syndrome, Cohesin

## Abstract

The data shown in this article are related to the research article entitled “Characterization of *Sgo1* expression pattern in developing and adult mouse” (Song et al., 2017) [3]. The article provides novel data on *Sgo1 gene* expression pattern utilizing Sgo1_LacZ_Knock in mouse line and immunohistochemistry in wild type mice. The data presents *Sgo1* expression pattern during development, and in post-developmental proliferative and quiescent tissue. The article describes following tissues: developing heart, neural tube, adult colon, cerebellum, cerebral cortex, liver, and testis.

## Specifications Table

**Table:** 

Subject area	*Biology*
More specific subject area	*Developmental biology, Cardiology, Gastroenterology, Ophthalmology, Neurology*
Type of data	*Immunohistochemistry, whole tissue X-gal stain, Western blot*
How data was acquired	Immunohistochemistry on wild type mouse tissue section X-gal stain on Sgo1-LacZ-Knock In mouse and section Western blot of protein extracted from corresponding mouse organs
Data format	*Raw and analyzed microscopy image*
Experimental factors	*All tissues were fixed in PFA and sectioned as paraffin or OCT block*
Experimental features	*The data shows Sgo1 expression in the developing heart, retina, central nervous system, liver, and adult mice colon*
Data source location	*Sgo1_LacZ Knock In mice were obtained from Wellcome Sanger Institute*
Data accessibility	*The data is with this article*

## **Value of the data**

• The data expands the currently limited Sgo1 expression pattern analysis during mammalian development and post-development, which can be a useful reference for other researchers.
• The data presents *Sgo1* expression in various tissue/cell types.
• Additional to the characteristic nuclear SGO1 localization in mitotic cells, the data describes non-nuclear localization of SGO1 in vivo.

## 1 Data

**Fig. 1:**
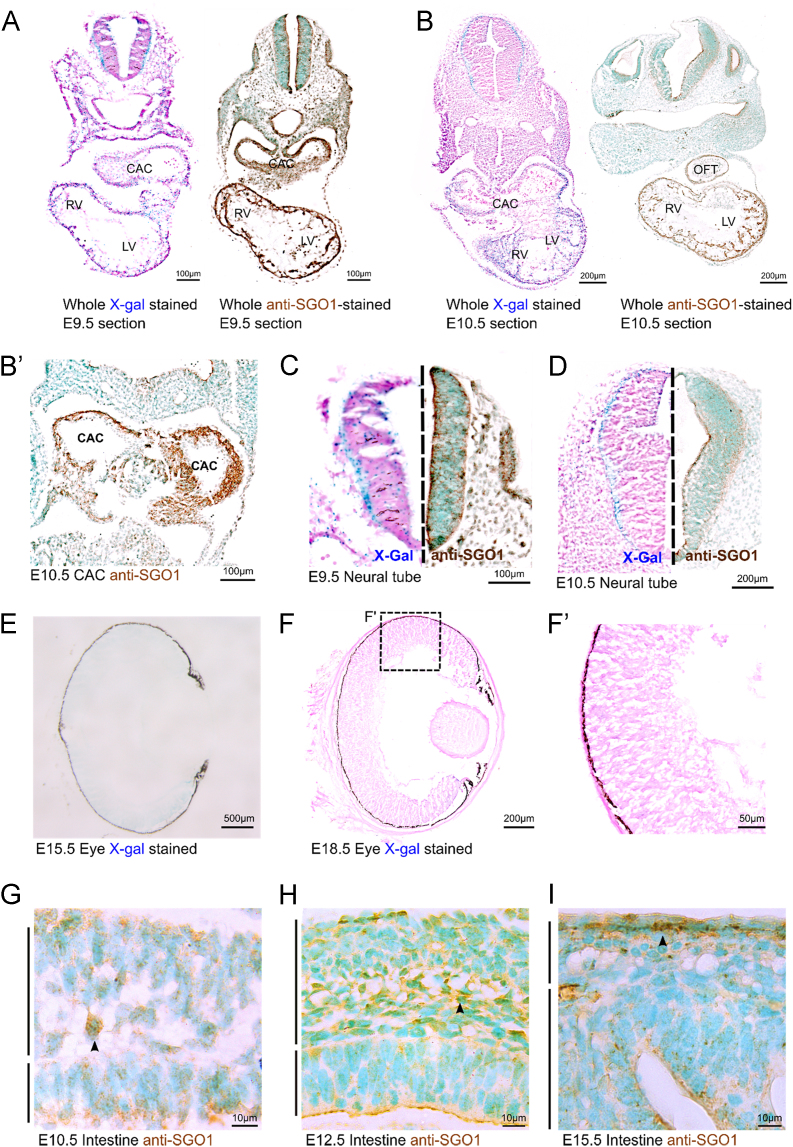
*Sgo1* expression in developing mice embryo. X-Gal stained sections were counterstained with Nuclear Fast Red and the anti-SGO1 immunostained sections were counterstained with methyl green. (A, B) A transverse section comparison between X-gal stained embyro versus anti-SGO1 immunostained embryo. (B′) A transverse section of E10.5 anti-SGO1 immunostain showed localization in CAC. (C, D) A comparison of Sgo1 expression in the neural tube between X-gal and anti-SGO1 immunostain at corresponding stages. (E) E15.5 retina showed minimal X-gal staining. (F, F′) E18.5 retina showed little X-gal stain. (G–I) Intestinal layer in corresponding stages. Lower aerobar indicates the intestinal epithelia. Upper aerobar indicates the intestinal mesenchyme. SGO1 localized to cells in the intestinal mesenchymal layers in E10.5 and E12.5. At E15.5, SGO1 localized to the cells at the border of the developing smooth muscle layer and serosa [black arrowheads]. LV: left ventricle, RV: right ventricle, OFT: outflow tract, CAC: common atrial chamber.

*Sgo1* expression pattern largely agreed between the X-Gal stain and immunostain at both E9.5 and E10.5. In both stages, *Sgo1* was expressed robustly in the heart and the neural tube (Fig. 1. A-B’). Closer observation of the neural tube revealed predominant localization of the X-Gal signal to its outer border whereas the immunostain marked the SGO1 protein in both the outer and inner borders (Fig. 1C and D). During the later stages of retinal development in E15.5 and E18.5, the X-gal signal diminished from faint to little signal (Fig. 1E–F′). SGO1 localized to cells in the intestinal mesenchymal layers in E10.5 and E12.5. At E15.5, SGO1 localized to the cells at the border of the developing smooth muscle layer and serosa (Fig. 1G–I).

**Fig. 2:**
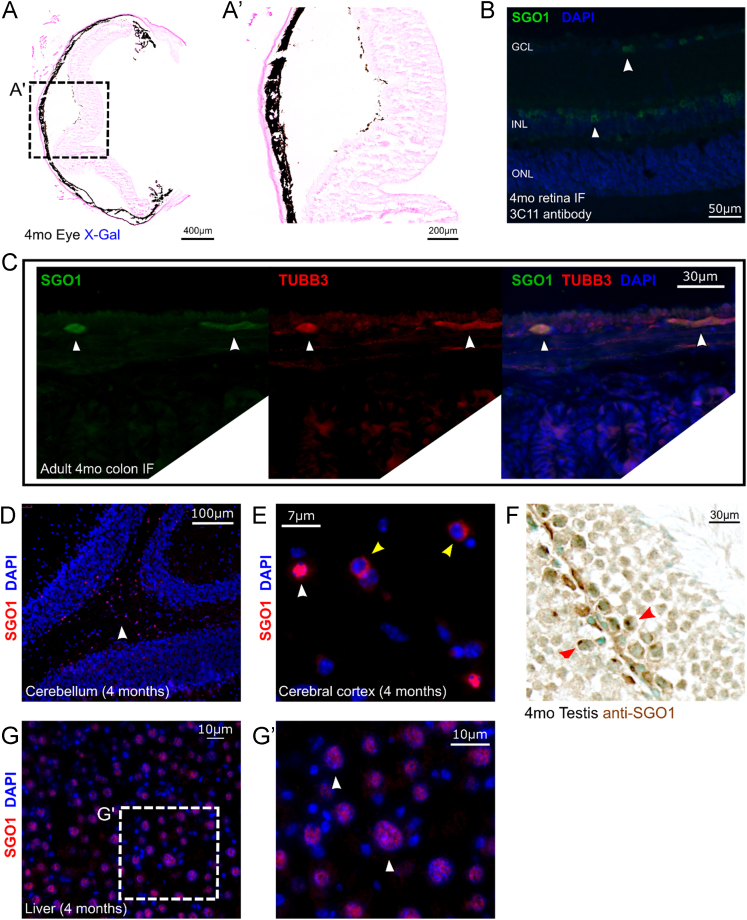
*Sgo1* expression in 4 month old (mo) adult mice tissues. (A, A′) 4mo retina showed no visibly recognizable X-Gal signal. (B) 4mo retina showed SGO1 protein localization in subset(s) of retinal neuronal cells using anti-SGO1 3C11 antibody. (C) SGO1 co-localized with TUBB3 positive intestinal ganglion cells in mouse colon. (D) SGO1 positive cells were largely localized in the white matter of the cerebellum [white arrowhead]. (E) SGO1 localization to the nuclear region of cells [white arrowhead] as well as cells with mutually exclusive localization of anti-SGO1 and DAPI signal [yellow arrowheads]. (F) 4mo mice testis showed punctate anti-SGO1 signal overlapping with methyl green nuclear stain in cells at the spermatogonium niche [red arrowhead]. (G, G′) Majority of hepatocytes showed nuclear SGO1 localization in normal 4mo wild type mice liver. (H) E15.5 mouse heart showed lack of SGO1 localization at the atrioventricular cushion.

X-Gal staining of 4 month old adult mice retina showed no discernable signal (Fig. 2A, A′). Anti-SGO1 immunostaining with 3C11 antibody (Abnova; H00151648-M01) showed SGO1 localization in select population of retinal neurons (Fig. 2B); also, shown with monoclonal antibody against SGO1 [3]. Furthermore, SGO1 localized in intestinal ganglion cells of adult mice colon that co-localized with anti-TUBB3, a marker for post-mitotic neurons (Fig. 2C). SGO1 in the cerebellum preferentially localized to the cells in the white matter region (Fig. 2. D). Intracellularly, we identified cells in the cerebral cortex that distinctly shows anti-SGO1 within the boundary of DAPI signal as well as cells which displayed anti-SGO1 signal outside the region positive for DAPI signal (Fig. 2E).

Spermatogonia, in the adult testis reside in the niche at the border of seminiferous tubule wall [1], displayed punctate anti-SGO1 signal, distinctly overlapping with DNA binding counterstain: methyl green. Hepatocytes in the liver are quiescent under normal conditions [2]. SGO1 localized diffusely throughout the nucleus in nearly all morphologically identifiable hepatocytes.

**Fig. 3:**
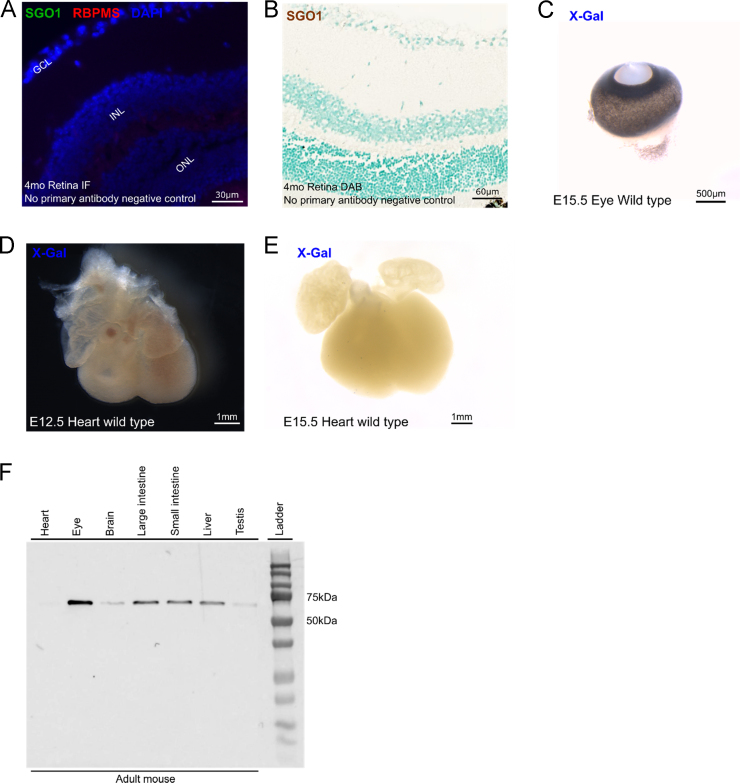
Negative controls for immunohistochemistry and X-Gal stain. Western blot of 4mo mice. (A) No primary antibodies control for anti-SGO1, RBPMS immunofluorescence stain did not show any unspecific signal. (B) No primary antibodies control for anti-SGO1 DAB-immunostain did not show any unspecific signal. (C–E) Wild type organs processed and incubated in X-Gal in the same conditions did not show any positive X-Gal signal. (F) Western blot performed with anti-SGO1 ab58023 on adult mice tissues.

No discernable false positive signals were observed under same experimental conditions in immunostains and X-gal stain (Fig. 3). Western blot of adult mouse tissues against SGO1 protein showed a band in the predicted region (59 kDa) of SGO1 protein size with no discernable unspecific binding (Fig. 3.). Refer to the following *Gene Expression Patterns* article for results and discussion [3].

## 2 Experimental design, materials and methods

### 2.1 Animal model

*Sgo1*^*+*/*LacZ*^ mice were received from Wellcome Trust Sanger Institute (WTSI). The *Sgo1*^*+*/*LacZ*^ mice have a flippase recognition target (FRT) flanked lacZ cassette inserted between exons 4 and 5 of the *Sgo1* gene in a C57Bl6/N strain. A splicing acceptor site precedes the FRT for inclusion of lacZ cassette into the transcript and following polyadenylation sequence, resulting in a truncated Sgo1 mRNA transcript, and protein. The crossings were made with *Sgo1*^*+*/*+*^ and *Sgo1*^*+*/*LacZ*^ and the appearance of vaginal plug was designated as Embryonic day (E) 0.5. Embryos at E9.5, E10.5, E12.5, E15.5, E18.5, and 4-month old adult mice, were collected for the experiment.

### 2.2 Dissection

Mice were sacrificed according to Canadian Council on Animal Care (CCAC) guidelines. The uterus was extracted and placed in cold phosphate buffer solution (PBS) during embryo extraction. The yolk sac of each embryo was used to genotype the mice using PCR. The samples were then left in PBS on ice for 5–10 min to reduce blood in the sample. The samples were incubated in 4% paraformaldehyde (PFA) at 4 °C with gentle rocking for; E9–E10.5: 5 min, E12–E15.5: 10 min, individual organs: 5 min. After rinsing in PBS to remove residual PFA, *Sgo1*^*+*/*LacZ*^ were permeated via rinse buffer (100 mM sodium phosphate, 2 mM MgCl_2_, 0.01% sodium deoxycholate, 0.02% NP-40) overnight at 4 °C with gentle rocking and the wild type samples for DAB staining were dehydrated to 70% ethanol gradually at this time point.

### 2.3 X-Gal staining

The X-Gal buffer and heterozygous embryos were warmed to 37 °C. X-Gal (5-bromo-4-chloro-3-indolyl-β-D-galactopyranoside; Multicell) dissolved in dimethyl-formamide (100 mg/ml) was then added to the X-Gal buffer (5 mM potassium ferricyanide, 5 mM potassium ferrocyanide in rinse buffer) immediately before use to a final X-Gal concentration of 1 mg/ml. The samples were incubated in this X-Gal staining solution for 24 h at 37 °C in total darkness with abundant movement for equal exposure to the staining solution. The samples were thoroughly washed in the rinse buffer on ice until the solution was completely free of yellow coloration from the X-gal buffer. They were then fixed in 4% PFA for minimum 48 h. The samples were gradually dehydrated to 70% ethanol for storage and imaging. For paraffin embedding, the samples were dehydrated gradually to 100% ethanol and incubated respectively in 50/50 ethanol/xylene, 2× 100% xylene, 50/50 xylene/paraffin for maximum 5 minutes each step and were finished with 2× paraffin at 60–62 °C for one hour. The samples were sectioned at 10 μm and counterstained with Nuclear Fast Red (NFR). For cryosectioning, the samples were submerged in a 1:1 ratio of 50% sucrose:Optimal Cutting Temperature (OCT) overnight and frozen in OCT with ethanol slurry on dry ice (OCT: Leica). The samples were equilibrated to the cryotome׳s working temperature (−20 °C), then sectioned at 12 μm to be counterstained with NFR.

### 2.4 Immunohistochemistry

DAB-immunostaining was performed according to the manufacturer׳s instruction (Thermo-Scientific; Ultravision LP Detection System). The wild type samples were sectioned at 10 μm. Antigen retrieval was performed with citrate buffer (10 mM sodium citrate, 0.05% Tween 20, pH 6.0) at 68 °C for 20 min and 0.3% Triton at room temperature for 30 min. The endogenous peroxidase activity was minimized with hydrogen peroxide incubation for 15 minutes for DAB staining. Two mouse monoclonal anti-SGO1 antibodies (abcam; ab58023 or abnova: M01_clone 3C11; 1/350 in 10% goat serum) were used separately and compared in representative slides to support the specificity of the SGO1 localisation. TUBB3 and RBPMS antibodies were acquired as the courtesy of Alyson Fournier׳s lab at Montreal Neurological Institute. The primary antibodies were targeted with rabbit anti-mouse IgG secondary antibody conjugated to horseradish peroxidase for DAB staining and goat anti-mouse Alexa 488 for fluorescent staining. DAB slides were counterstained with methyl green and mounted with Permount medium. Fluorescent slides were mounted with DAPI incorporated anti-fade medium.

### 2.5 Western blot

Protein lysates were extracted from the corresponding tissues with RIPA buffer and concentration was quantified using BCA assay. The protein extracts were reduced with 5% end concentration of beta-merceptoethanol and denatured at 95 °C for 5 min before the SDS-PAGE. After the transfer, the nitrocellulose blot was blocked with 5% bovine serum albumin and incubated with anti-SGO1 ab58023 antibody followed by goat anti-mouse HRP conjugated antibody. The blot was developed with ECL reagent.
