# Supplementary material for: Dataset of Sgo1 expression in cardiac, gastrointestinal, hepatic and neuronal tissue in mouse
Source: Data Brief. 2017 Jul 1;13:731–7. doi: 10.1016/j.dib.2017.06.046 (PMC5512188; doi:10.1016/j.dib.2017.06.046)
Supplement: Supplementary file 1 — Supplementary material [file mmc1.docx]

Montreal, 30/5/2017

Prof. D. Stainier

Editor, Gene Expression Patterns

- by electronic submission -

Dear Didier:

After the publication of our original article ‘Characterization of Sgo1 expression in developing and adult mouse’ in GEP in April, we are now resubmitting the revised Data in Brief companion article.

We have given careful consideration to the Reviewer’s suggestions and think that this dataset now provides an interesting complement to the Original Article.

This manuscript is not under consideration elsewhere, and none of the manuscript’s results have been published previously. All authors have read and approved the manuscript.

We wish to confirm that there are no known conflicts of interest associated with this publication and there has been no significant financial support for this work that could have influenced its outcome.

Thank you for receving our revised DIB manuscript. We look forward to your response.

Yours sincerely,


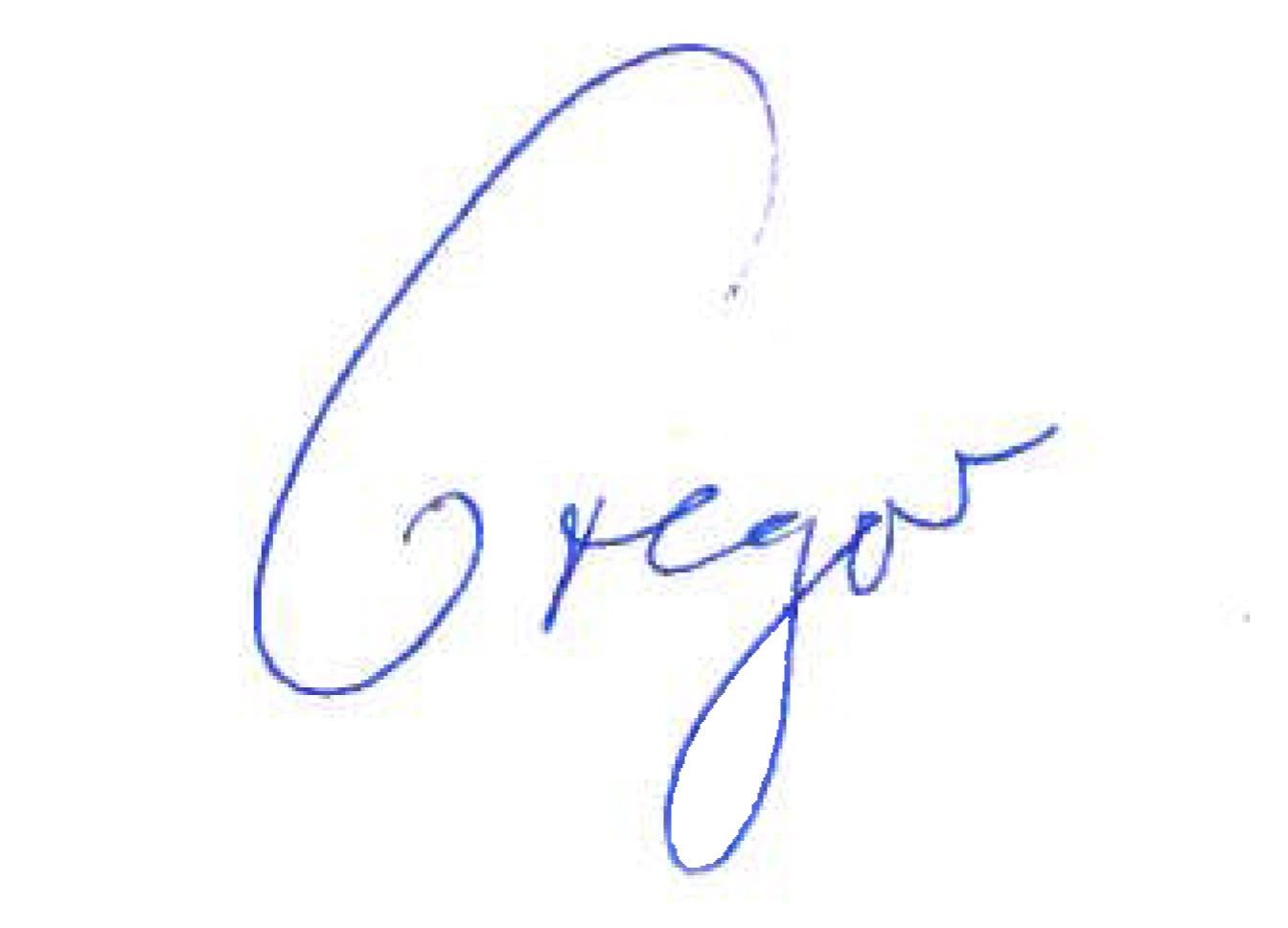


Gregor Andelfinger, M.D., FRCPC

Head, Cardiovascular Genetics Research Laboratory
